# Engineering a Cold-Active Cellulase Complex with a Novel Mushroom Cellobiohydrolase for Efficient Biomass Saccharification and Juice Flavor Optimization

**DOI:** 10.3390/jof12040276

**Published:** 2026-04-10

**Authors:** Jiaqi Yang, Youran Shao, Ying Wang, Ming Gong, Bing Li, Hongyu Chen, Caizhen Wang, Yan Li, Xiang Zhou, Gen Zou

**Affiliations:** 1 National Key Laboratory for Development and Utilization of Forest Food Resources, School of Forestry and Biotechnology, Zhejiang A&F University, Hangzhou 311300, China; jqyang@stu.zafu.edu.cn; 2 National Engineering Research Center of Edible Fungi, Key Laboratory of Applied Mycological Resources and Utilization, Ministry of Agriculture, Institute of Edible Fungi, Shanghai Academy of Agricultural Sciences, Shanghai 201403, China; yrshao@saas.sh.cn (Y.S.); wangyingmush@saas.sh.cn (Y.W.); minggong@saas.sh.cn (M.G.); libing@saas.sh.cn (B.L.); chenhongyu20128@163.com (H.C.); liyan03@saas.sh.cn (Y.L.); 3 College of Life Sciences, Shanghai Normal University, Shanghai 200234, China; 13524869286@163.com

**Keywords:** cold-active cellobiohydrolase, saccharification, product inhibition, pomace, biorefinery

## Abstract

Cold-active cellulases are highly desirable for temperature-sensitive biomass valorization and food processing, yet they remain scarce in conventional industrial fungal platforms. In this study, a novel cold-induced cellobiohydrolase, *Vv*CBHI-II, was mined from the mushroom *Volvariella volvacea* and successfully engineered into the industrial workhorse *Trichoderma reesei* via site-specific homologous replacement. Structural homology modeling revealed that the substitution of the flexible B3 loop with a β-sheet creates a more open substrate-binding cleft in *Vv*CBHI-II. Consequently, the purified *Vv*CBHI-II exhibited robust endoglucanase-like characteristics with superior catalytic efficiency on amorphous cellulose. At 10 °C, the engineered cellulase complex demonstrated an 8.1-fold increase in filter paper activity compared to the wild-type strain. Mechanistic structural analyses indicated that the open cleft architecture elongates and weakens the hydrogen-bonding network with the cellobiose product, facilitating rapid product dissociation and alleviating severe cold-induced product inhibition. In practical applications, the engineered cold-active enzyme complex exhibited an exceptional saccharification capacity on natural pear pomace at 10 °C. Furthermore, when applied to simulated fruit juice processing, it significantly maximized the extraction yield, elevated the sweetness response, and substantially mitigated undesirable bitterness and astringency. This study elucidates the structural-functional paradigm of cold-adapted cellobiohydrolases and provides a promising strategy for formulating highly efficient, energy-saving biocatalysts for the food and biorefinery industries.

## 1. Introduction

The waste generated by the fruit and vegetable processing industry constitutes the primary source of residues within the entire food processing sector [1]. Among the many factors contributing to the global environmental burden in recent years, the profound impact of fruit and vegetable waste has been identified as a major challenge [2]. For instance, the proportion of waste materials produced during most fruit and vegetable processing is typically high [3]. Due to the inherent biological characteristics of these agricultural products, substantial losses inevitably occur during industrial handling and processing stages, including sorting, washing, peeling, and coring [4]. Furthermore, their rich endogenous enzyme content and susceptibility to enzymatic browning render waste generation largely unavoidable during processing [5,6]. Compounding this issue, their high moisture content makes them highly perishable under suboptimal storage conditions, thereby exacerbating total food loss [7]. Although recent efforts have attempted to valorize these residues into high-value bioproducts—such as sustainable alternatives to animal leather—these applications have not yet achieved mainstream commercialization [8]. Currently, employing enzymatic pretreatment to minimize waste generation remains the predominant strategy [9,10]. This is primarily because enzymatic technology serves as a core approach to significantly enhance extraction efficiency, improve product stability, and upgrade sensory quality [11,12,13].

Carbohydrate-active enzymes—particularly cellulases, hemicellulases (e.g., xylanases and mannanases), and pectinases—can significantly enhance juice yield, promote turbidity stability and clarification, and liberate bound flavor substances and nutritional compounds through the synergistic depolymerization of the complex polysaccharide networks inherent in plant cell walls [11,14]. A quintessential example of this processing bottleneck is observed in pear juice production. The rigid structure of highly lignified stone cells within pear tissue severely limits mechanical extraction efficiency and negatively impairs the sensory mouthfeel of the final product [15]. Consequently, enzymatic intervention is virtually indispensable for dismantling these cellular barriers to maximize juice yield and improve texture.

However, conventional commercial enzyme cocktails, which are predominantly derived from fungal systems such as *Aspergillus* or *Trichoderma* species [16,17,18], typically exhibit relatively high optimal temperatures (often above 40 °C), and their hydrolytic activities are severely constrained under low-temperature conditions [19]. This inherent limitation not only restricts their application in temperature-sensitive processes, such as the processing of heat-sensitive fruit and vegetable juices, but may also lead to increased energy consumption and undesirable flavor loss [20]. Since preserving the fresh aroma, heat-sensitive nutrients, and overall quality of premium cold-pressed juices (such as pear juice) is paramount, the industry urgently requires efficient low-temperature alternatives. Furthermore, the enzymatic degradation of plant cell walls not only increases sugar extraction but also profoundly influences the release of flavor precursors and phenolic compounds, which dictate the delicate balance of sweetness, bitterness, and astringency [20].

Consequently, the discovery and development of novel cold-active glycoside hydrolases capable of maintaining robust catalytic efficiency at ambient or low temperatures have emerged as a pivotal research direction in the juice processing industry [21]. Cold-active cellulases are predominantly derived from psychrophilic or psychrotolerant microorganisms, achieving highly efficient catalysis at low temperatures through molecular structural adaptations, such as enhanced flexibility, reduced salt bridges, and fewer hydrophobic interactions [22,23]. In recent years, researchers have increasingly prospected for these enzymes from non-traditional sources, including insects, marine organisms, and fungi inhabiting specialized [21]. Besides juice processing, the industrial applications of cold-active cellulases have progressively expanded from traditional textile [24,25] and detergent sectors [26] to temperature- and energy-sensitive fields, including food processing, animal feed and green biorefineries [17,20,27].

*Volvariella volvacea*, a typical high-temperature edible mushroom, presents postharvest biological characteristics that offer novel clues for the discovery of unique cold-active enzymes [28]. During low-temperature storage, its fruiting bodies are highly susceptible to a softening phenomenon known as cryogenic autolysis, a process intricately associated with the low-temperature-induced upregulation of endogenous cellulases and glycoside hydrolases [29]. Strikingly, the enzyme proteins within this cold-induced system have evolved the intrinsic capacity to sustain robust activity in cold environments to accommodate their physiological autolysis requirements. This indicates that *V. volvacea* harbors a specialized glycoside hydrolase system capable of maintaining high activity under low-temperature conditions. Given their specific induction by cold stress, these enzymes likely possess distinctive catalytic properties and substantial application potential [17]. However, current investigations into the functions of these enzymes, particularly regarding their specific roles during mycelial growth and fruiting body development, remain inadequate. Consequently, their cold-adapted catalytic mechanisms and prospective industrial value warrant further in-depth exploration.

Therefore, the present study aimed to achieve the heterologous expression and functional characterization of *Vv*CBHI-II, a cold-induced glycoside hydrolase from *V. volvacea*, in the industrial workhorse *T. reesei*. Although a few commercial cold-active enzyme preparations are available from leading manufacturers (e.g., Novozymes), they are predominantly formulated as complex enzyme cocktails. This complexity makes direct biochemical comparisons with a single, newly discovered enzyme scientifically challenging at the fundamental characterization stage. Furthermore, the continuous discovery of novel cold-active enzymes from diverse biological sources—such as edible mushrooms—is urgently needed to enrich the industrial enzyme toolbox and provide unique catalytic advantages for specific recalcitrant substrates [16]. Consequently, while this work currently focuses on the fundamental properties of the individual enzyme *Vv*CBHI-II, its overarching objective remains highly applied. To bridge the critical gap between basic discovery and future commercial scale-up, *T. reesei* was explicitly selected as the expression host over simple bacterial systems. The primary objective was to develop a novel cold-active glycoside hydrolase preparation tailored for fruit juice processing. By systematically elucidating its enzymatic properties and evaluating its hydrolytic performance on natural lignocellulosic substrates, this work provides a novel enzyme source and a robust theoretical foundation for formulating highly efficient, energy-saving, and premium cold-active enzyme cocktails. Furthermore, this study explores the promising application potential of *Vv*CBHI II in low-temperature juice extraction, specifically targeting recalcitrant substrates like pear pulp, while concurrently opening new avenues for the valorization of functional enzymes derived from *V. volvacea*.

## 2. Materials and Methods

### 2.1. Strains and Culture Conditions

Competent cells of *Escherichia coli* and *Agrobacterium tumefaciens* AGL1 were procured from Weidi Biotechnology Co., Ltd. (Shanghai, China) and cultured in Luria–Bertani (LB) medium (10 g/L tryptone, 5 g/L yeast extract, and 10 g/L NaCl) [30]. These strains were utilized as the cloning host and the mediator for the genetic transformation of wild-type *T. reesei* Rut-C30 (maintained in our laboratory), respectively [30]. *T. reesei* Rut-C30 was cultivated on potato dextrose agar (PDA) medium (200 g/L potato infusion, 20 g/L glucose) at 28 °C under illumination. For the heterologous expression of *vvcbh1*, the recombinant *T. reesei* was inoculated into a Sabouraud dextrose broth (SDB, 0.4 g/L glucose, 0.1 g/L yeast extract, and 0.1 g/L peptone) at a ratio of 1:10 (*v*/*v*) and incubated for 48 h. Subsequently, the seed culture was transferred into a fermentation medium supplemented with 3% (*w*/*v*) microcrystalline cellulose, 2% (*w*/*v*) wheat bran, and 0.3% (*w*/*v*) peptone, and cultured for 7 days [31].

### 2.2. Plasmid Construction

The expression vector pxbthg-30, maintained in our laboratory, was utilized as the cloning backbone. The *vvcbhI-II* gene derived from *V. volvacea* (GenBank accession number: AAT64007.1) was codon-optimized and synthesized by Synbio Technologies (Suzhou, China) (Tables S1 and S2) (Data S1). To generate the linearized vector, the plasmid was double-digested with *Bam*HI and *Hind*III restriction enzymes. Specific primers (Vvcbh1F/R, TtrpC1F/R, and Pcbh1F/R) were designed to amplify the full-length *vvcbhI-II* sequence, the TrpC1 fragment, and the Pcbh1 fragment, utilizing the synthesized *vvcbhI-II* gene, the pxbthg-30 plasmid, and *T. reesei* genomic DNA as templates, respectively (Table S1). The assembled recombinant plasmid was transformed into *E. coli* TOP10 competent cells, and positive single clones were selected and verified via colony PCR. Subsequently, the validated plasmids were extracted and transformed into *A. tumefaciens* AGL1. Positive monoclonal transformants were further confirmed via PCR and DNA sequencing [32]. A 6× His-tag was fused to the C-terminus of the *Vv*CBHI-II sequence to facilitate specific recognition by the corresponding antibody in downstream analyses.

### 2.3. Strain Construction and Protein Expression

A 1-mL suspension (OD_600_ = 0.8) of *A. tumefaciens* was mixed with 1 mL of *T. reesei* Rut-C30 conidia (10^7^ conidia per mL), which were harvested using a 0.02% (*v*/*v*) Tween-80 solution. The mixture was co-incubated on a rotary shaker at 25 °C and 80 rpm for 20 min, followed by co-cultivation in the dark at 28 °C for 2 days. The spores from the co-culture plate were then eluted with 2 mL of 0.02% Tween-80, and 300-μL aliquots were spread onto PDA selection plates supplemented with 30 μg/mL hygromycin B and 300 μg/mL cefotaxime. After approximately 3 days of incubation, the germinated colonies were selected and transferred to fresh PDA selection plates for secondary screening. Genomic DNA of the putative transformants was extracted and verified via PCR (Table S1). Following the *Agrobacterium*-mediated transformation, PCR analysis was performed to screen and select the positive transformants with site-specific homologous recombination (HR) integration at the *trcbh1* locus (Table S1, Figure S1).

For protein expression, conidia from both the wild-type and the positive transformants were harvested using 0.02% Tween-80 and inoculated at a 1:10 (*v*/*v*) ratio into 30 mL of SDB. These cultures were incubated at 28 °C and 200 rpm for 7 days to generate seed cultures. Subsequently, the fresh seed cultures were transferred into a fermentation medium at a 1:10 (*v*/*v*) inoculum ratio and cultivated for an additional 7 days. The fermentation broth was then centrifuged at 12,000× *g* and 4 °C for 5 min, and the resulting supernatant was collected as the crude cellulase complex. The protein expression profile of the crude enzyme was evaluated using sodium dodecyl sulfate-polyacrylamide gel electrophoresis (SDS-PAGE) and Western blotting. Specifically, an anti-6× His-tag antibody (Abclone, Wuhan, China) was employed to specifically detect the 6× His-tag fused to the recombinant protein [31].

The recombinant *Vv*CBHI-II was purified from the fermentation supernatant utilizing His-tag affinity chromatography (Ni-NTA). The purification procedure was conducted strictly following the established protocol previously described for the expression and purification of *Vv*CBHI-I and *Tr*CBHI in our prior study [17]. The purified enzyme fractions were subsequently desalted, concentrated, and stored in a 0.05 M citrate-sodium citrate buffer (pH 5.0) for downstream specific activity assays.

### 2.4. Cellulase Activity Assays

Activities on filter paper (FPase), phosphoric acid-swollen cellulose (PASC), Avicel PH-101 (DuPont, Copenhagen, Denmark), xylanase (xylan from beechwood, Yuanye Biotech. Co., Ltd., Shanghai, China) and CMC-Na (CMCase) were determined utilizing the 3,5-dinitrosalicylic acid (DNS) method [33]. The principle of this assay relies on quantifying the amount of reducing sugars released from the substrates following enzymatic hydrolysis. In accordance with the internationally recognized standard guidelines for cellulase activity evaluation [33], the concentration of total reducing sugars was calculated and expressed as glucose equivalents utilizing a standard glucose calibration curve. The reaction mixture consisted of 60 µL of citrate-sodium citrate buffer (0.05 M, pH 5.0) and 20 µL of 10-fold diluted crude enzyme solution. Standard Whatman filter paper discs (0.5 cm in diameter), 60 µL of CMC-Na or PASC solution were employed as substrates for FPase, PASCase and CMCase activities, respectively. The mixtures were incubated at 10 °C and 50 °C for 60 min (for FPase and PASCase) and 10 min (for CMCase). Subsequently, 120 µL of DNS reagent was added, and the reaction was heated in a boiling water bath for 10 min for color development. After cooling, deionized water was added to reach a final volume of 1 mL, and the absorbance was measured at 540 nm using a microplate reader. Under these conditions, one international unit (IU) of enzyme activity was defined as the amount of enzyme required to release 1 μmol of glucose per minute.

Furthermore, β-glucosidase and cellobiohydrolase activities were evaluated based on the *p*-nitrophenol (*p*NP) method. A commercial kit from Boxbio Science & Technology Co., Ltd. (Beijing, China) was used for determining β-glucosidase activity, and a kit from Grace Biotechnology Co., Ltd. (Suzhou, China) was utilized for cellobiohydrolase activity. Following incubation at 10 °C and 50 °C for 30 min, the absorbance of the reaction mixtures was recorded at 405 nm. One unit (U) of these enzyme activities was defined as the amount of enzyme that catalyzed the liberation of 1 micromole of *p*NP per minute under the specified assay conditions [17]. All enzyme activity determinations included three or five replicates, and statistical analyses were performed using a two-tailed Student’s *t*-test (* *p* < 0.05, ** *p* < 0.01, *** *p* < 0.001).

### 2.5. Saccharification of Natural Lignocellulosic Biomass

Fresh Dangshan pears were procured from a local market in Fengxian District (Shanghai, China). Following three times homogenization (15 s) with a homogenizer (TENLIN-C, Jiangsu Tianling Instrument Co., Ltd., Yancheng, China), the pomace particles were separated and collected via filtration through a double-layer cheesecloth, and subsequently dried to a constant weight in an oven at 60 °C. The dried biomass was then milled and sieved to obtain granules of <0.5 mm in diameter. To eliminate residual soluble sugars and impurities, the solid particles were washed thoroughly with deionized water three times, re-dried to a constant weight, and stored in a sealed container for future use.

For the saccharification assay, 0.02 g of the dried pomace particles was suspended in 2 mL of 0.05 M citrate-sodium citrate buffer (pH 4.8). Varying volumes of the crude cellulase complex (fermentation supernatant was quantified to 10 FPU/mL) produced by the transformants and the wild-type Rut-C30 under optimal fermentation conditions were added to the mixture. The enzymatic hydrolysis was conducted in a shaking incubator at 10 °C for 12 h. To quantitatively evaluate the degradation capability of the respective enzyme preparations on the insoluble matrix of Dangshan pear, the mass of the pomace particles was recorded and compared before and after hydrolysis. Concurrently, to visually assess the structural disruption of the substrate surface induced by enzymatic degradation, a stereomicroscope (15× magnification, 15.33 mm field of view diameter) was employed to observe the morphological changes, and the average particle area was computed using ImageJ software (https://imagej.net/, accessed on 1 November 2025). To precisely quantify the strictly monomeric glucose liberated during the saccharification process, the glucose concentration in the hydrolysate was determined utilizing a highly specific Glucose Oxidase–Peroxidase (GOPOD) Assay Kit (Beijing Leagene Biotech Co., Ltd., Beijing, China). Furthermore, to investigate the low-temperature hydrolytic efficacy of the cellulase preparation secreted by the transformant and the wild-type Rut-C30 strain on juice flavor precursors, an additional enzymatic assay was conducted. The cellulase preparations of H2 and WT were loaded at varying volumes of 2–100 μL per 50 mL unfiltered pear juice. Crucially, prior to addition, both enzyme preparations were strictly quantified and standardized to an equivalent concentration of 10 FPU/mL to ensure an impartial comparison. The enzymatic hydrolysis was carried out at 10 °C for 24 h. Subsequently, the flavor response values of the resulting hydrolysates were quantitatively evaluated utilizing an E-tongue system (INSENT SA402B, Intelligent Sensor Technology, Inc., Kanagawa, Japan).

## 3. Results and Discussion

### 3.1. Genome Mining and Selection of Target GH7 CBH Genes

**Figure 1:**
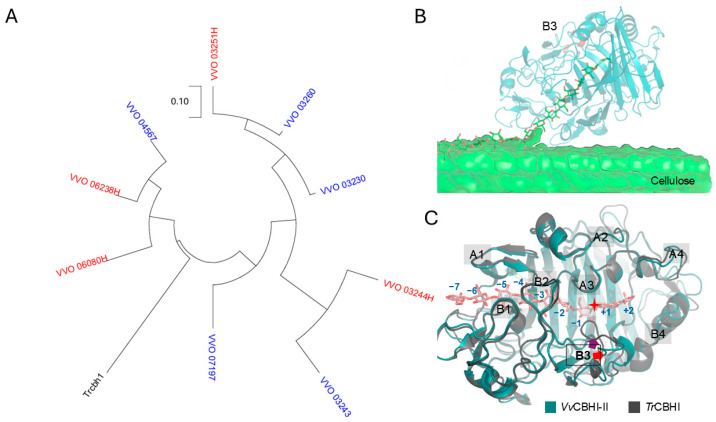
Genome mining, phylogenetic analysis, and structural modeling of *Vv*CBHI-II. (**A**) Phylogenetic tree of GH7 family cellobiohydrolases revealing the evolutionary relationship of *V. volvacea* candidates and *T. reesei Tr*CBHI. Blue: Putative cold-adapted cellobiohydrolases associated with the cold-induced autophagy stage. Red: Putative cellobiohydrolases expressed during the primordium/fruiting body development stage. (**B**) Homology model of the *Vv*CBHI-II catalytic domain interacting with a cellulose chain, highlighting the position of the B3 loop. (**C**) Superposition of the 3D structures of *Vv*CBHI-II and *Tr*CBHI (PDB: 4C4C), emphasizing the structural variations in the flexible loops (A1–A4 and B1–B4) surrounding the active site cleft. Red cross-star: active site cleft; Red arrow: *Vv*CBHI-II B3; Purple arrow: *Tr*CBHI B3.

Due to the limitations of the early genome assembly of *V. volvacea*, the glycoside hydrolase family 7 (GH7) cellobiohydrolase (CBH) genes were not comprehensively annotated. Through re-annotation and expression profiling analysis, a total of nine GH7 family enzymes were identified (Table S2 and Data S1). Among these, four proteins (VVO_03251, VVO_06238, VVO_06080, and VVO_03244H) are expressed during the fruiting body development stage, whereas five glycosylhydrolases (VVO_03260, VVO_03230, VVO_04567, VVO_07197, and VVO_03243) are specifically upregulated by cold stress. Phylogenetic analysis (Figure 1A) revealed no distinct evolutionary divergence between the cold-induced and development-associated proteins. Specifically, the cold-active VVO_04567, along with the mesophilic VVO_06238 and VVO_06080, clustered together with *Tr*CBHI from *Trichoderma reesei*. Meanwhile, our previously characterized VVO_03230 (designated as *Vv*CBHI-I) clustered with VVO_03251 and VVO_03260. To further explore the functional diversity of cold-active CBHs, another highly cold-induced gene, VVO_07197, which is located in a phylogenetically distinct clade, was selected as the new target. A primary structural distinction between VVO_07197 and *Vv*CBHI-I lies in the absence of a carbohydrate-binding module (CBM) in the former and its presence in the latter. Consequently, VVO_07197 was named *Vv*CBHI-II.

By employing homology modeling based on the crystal structure of *Tr*CBHI bound to cellononaose (PDB 4C4C), the 3D model of the *Vv*CBHI-II catalytic domain during cellulose hydrolysis was constructed (Figure 1B). The region highlighted in red corresponds to the B3 loop, which exhibits poor sequence homology with its counterpart in *Tr*CBHI. In *Tr*CBHI, the B3 loop is a critical structural element dictating its catalytic activity and thermostability. Strikingly, in *Vv*CBHI-II, a portion of the B3 loop is replaced by a *β*-sheet (Figure 1C), resulting in a more open and accessible substrate-binding cleft. Conversely, other previously characterized loops in *Tr*CBHI are highly conserved in the 3D structure of *Vv*CBHI-II.

Furthermore, this structural variation in the B3 loop holds significant functional implications. In typical GH7 CBHs such as *Tr*CBHI, the active site is largely enclosed by a series of flexible loops (e.g., A1–A4 and B1–B4) that form a long substrate-binding tunnel [34]. While this tunnel architecture is essential for the processive hydrolysis of crystalline cellulose, it frequently results in severe product inhibition by cellobiose. Previous studies have demonstrated that deletions or structural modifications in the B3 loop of *Tr*CBHI can substantially reduce product and alter its kinetic profile, yielding higher *kcat* and *Km* values on soluble substrates, which resembles a more endoglucanase-like (EG-like) behavior [35]. Consequently, the more open and accessible substrate-binding cleft in *Vv*CBHI-II, resulting from the replacement of the B3 loop with a *β*-sheet, strongly suggests that this novel enzyme may exhibit enhanced tolerance to product inhibition and distinct catalytic flexibility during the degradation of complex lignocellulosic biomass.

### 3.2. Heterologous Expression of VvCBHI-II

**Figure 2:**
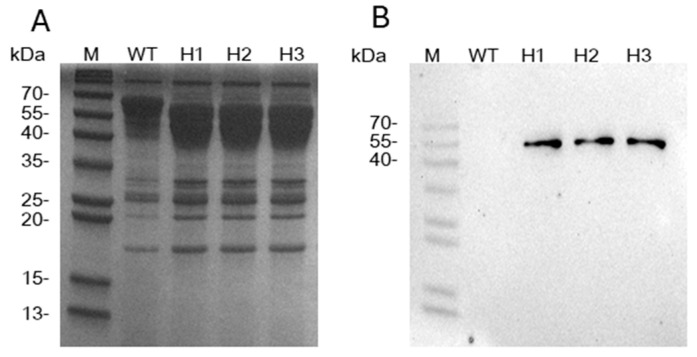
Heterologous expression of *Vv*CBHI-II in *T. reesei*. (**A**) SDS-PAGE analysis of the secreted proteins from the wild-type (WT) strain and homologous recombination transformants (H1–H3). M: marker. (**B**) Western blot analysis for the specific detection of the recombinant *Vv*CBHI-II protein in the culture supernatants.

The full-length *Vv*CBHI-II comprises 458 amino acids, including an *N*-terminal signal peptide (residues 1–20) and a glycoside hydrolase (GH) family 7 catalytic domain (residues 21–455). To investigate whether *Vv*CBHI-II possesses superior enzymatic properties, its heterologous expression was performed. To achieve stable heterologous expression, the constructed recombinant plasmids were integrated into the *T. reesei* genome via site-specific homologous recombination (HR). Three transformants with precise homologous replacement of *vvcbhI-II* at the endogenous *trcbh1* locus (designated as H1–H3) were selected for subsequent evaluation. The successful secretion of the target protein was evaluated via SDS-PAGE (Figure 2A) and Western blot (Figure 2B) analyses. A distinct and specific target protein band for *Vv*CBHI-II was successfully detected at approximately 55 kDa in the culture supernatants of these HR strains.

**Figure 3:**
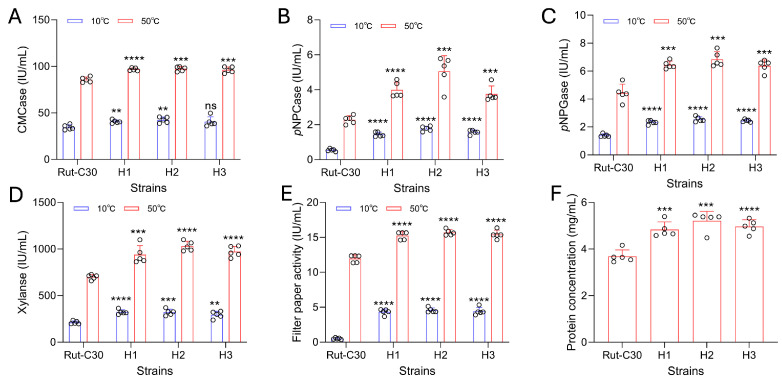
Enzymatic characterization of the crude cellulase complexes secreted by the recombinant *T. reesei* strains. Comparison of (**A**) CMCase, (**B**) *p*NPCase, (**C**) *p*NPGase, (**D**) xylanase, and (**E**) FPase between the wild-type (Rut-C30) and the homologous replacement transformants (H1–H3) at 10 °C and 50 °C. (**F**) Total extracellular protein concentrations in the fermentation broths. Data are presented as the mean ± standard deviation of replicates. Asterisks indicate statistical significance (ns *p* > 0.05, ** *p* < 0.01, *** *p* < 0.001, **** *p* < 0.0001).

Assays for CMCase, *p*NPCase, *p*NPGase, xylanase, and FPase activities were systematically conducted. At 50 °C, all evaluated enzymatic activities of the H1–H3 strains were significantly higher than those of the wild-type strain. Remarkably, at 10 °C, with the exception of the CMCase activity in strain H3—which showed no significant difference from the wild-type—all other HR strains consistently exhibited significantly enhanced activities compared to the wild-type strain across all assays (Figure 3A–E). This might be due to the fact that the CMC-Na solution, under low-temperature conditions, lacks sufficient fluidity, resulting in excessively high viscosity. This, in turn, restricts the contact between the enzyme and the substrate and also limits the release of the product. Notably, at 10 °C, the *p*NPCase activity of the transformants was 1.6- to 2.3-fold higher than that of the wild-type, while the FPase activity showed a striking 7.6- to 8.1-fold increase. Conversely, at 50 °C, apart from the *p*NPCase activity (which increased by 1.4-fold in transformant H2), the enhancements in other enzymatic activities were relatively modest, ranging from 10.8% to 52.7%. This overall activity improvement might be correlated with the higher total extracellular protein concentrations in the fermentation supernatants of H1–H3 (Figure 3F). Given that the secreted extracellular proteins in the industrial *T. reesei* system are predominantly comprised of lignocellulose-degrading enzymes, this total protein concentration serves as a direct indicator of the recombinants’ productivity. The productivity of the engineered strains reached up to 4.8–5.2 mg/mL, which increased by 31.0% to 41.3%. This observation is in good agreement with the SDS-PAGE profiles (Figure 2A), where nearly all secreted protein bands of the recombinant transformants appeared much denser than those of the wild-type. Therefore, it remains to be elucidated whether the ubiquitous enhancement of enzymatic activities in the recombinant transformants is driven by the overall increase in extracellular secreted proteins or explicitly dictated by the functional substitution of *Tr*CBHI with *Vv*CBHI-II.

### 3.3. Substrate Specificity of the Heterologously Expressed VvCBHI-II

To better elucidate the enzymatic properties of *Vv*CBHI-II, the recombinant *Vv*CBHI-II, along with the previously characterized *Vv*CBHI-I and *Tr*CBHI, were purified from their respective overexpressing *T. reesei* strains utilizing His-tag affinity chromatography. For comparative biochemical analysis, the previously characterized *Vv*CBHI-I (a naturally occurring paralog with a CBM from *V. volvacea*) and the industrial standard *Tr*CBHI, which were purified using the exact same protocols in our previous study [17] were also included as references. A comprehensive substrate specificity analysis of these three purified enzymes was conducted using diverse cellulosic substrates, including amorphous PASC, microcrystalline Avicel, CMC-Na, filter paper, and *p*NPC (Table 1).

As summarized in Table 1, *Vv*CBHI-II exhibited remarkable hydrolytic performance on amorphous cellulose, demonstrating the highest specific activity toward PASC (3.321 ± 0.056 U/mg), which was approximately 2.7-fold and 15.2-fold higher than those of *Vv*CBHI-I and *Tr*CBHI, respectively. Furthermore, *Vv*CBHI-II displayed notably elevated activity against the soluble substrate CMC-Na (0.234 ± 0.024 U/mg), surpassing *Vv*CBHI-I and *Tr*CBHI by 2.9-fold and 5.6-fold. This elevated CMCase activity strongly corroborates our earlier structural prediction that the replacement of the B3 loop with a *β*-sheet creates a more open substrate-binding cleft, conferring endoglucanase-like (EG-like) characteristics to *Vv*CBHI-II.

Regarding the synthetic substrate and filter paper, *Vv*CBHI-II also outperformed the other two counterpart enzymes, showing the highest specific activities toward *p*NPC (0.083 ± 0.011 U/mg) and filter paper (0.356 ± 0.038 U/mg). Conversely, its hydrolytic activity on highly crystalline Avicel (0.136 ± 0.016 U/mg) was slightly lower than that of *Vv*CBHI-I (0.265 ± 0.023 U/mg) and *Tr*CBHI (0.231 ± 0.023 U/mg). This distinct substrate preference indicates that while *Vv*CBHI-II possesses superior catalytic efficiency on amorphous and soluble substrates, its capacity to degrade highly crystalline cellulose is partially compromised. This trade-off is a classic feature of cellobiohydrolase evolution and engineering, where a wider and more flexible binding cleft enhances the turnover rate on easily accessible substrates but may weaken the tight, processive binding required for crystalline cellulose depolymerization [17,31].

### 3.4. VvCBHI-II Contributes to High Catalytic Activity Under Low-Temperature Conditions

**Figure 4:**
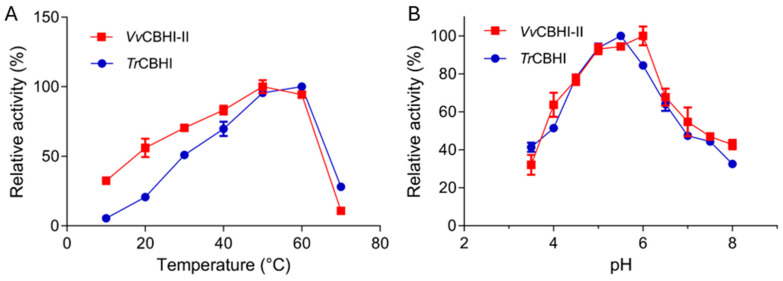
Biochemical properties of the purified recombinant *Vv*CBHI-II and *Tr*CBHI. (**A**) Effect of temperature on the relative enzymatic activities. (**B**) Effect of pH on the relative enzymatic activities. Both enzymes were assayed using *p*NPC as the substrate. The maximum activity obtained for each enzyme was defined as 100%. Data are expressed as the mean ± standard deviation of replicates.

To explicitly determine the environmental adaptability of the newly identified enzyme, the temperature and pH profiles of the purified recombinant *Vv*CBHI-II and *Tr*CBHI were comparatively evaluated (Figure 4). As depicted in Figure 4A, although both enzymes exhibited optimal catalytic performance at approximately 50 °C, *Vv*CBHI-II displayed significantly superior relative activity under low-temperature conditions (10–40 °C). Notably, at 10 °C and 20 °C, *Vv*CBHI-II retained a substantially higher percentage of its maximal activity compared to *Tr*CBHI, which severely lost its catalytic capacity in cold environments. Meanwhile, both enzymes shared a highly similar acidic pH profile, with an optimum pH ranging from 5.0 to 6.0 (Figure 4B). These biochemical properties definitively characterize *Vv*CBHI-II as a robust cold-active cellobiohydrolase.

**Figure 5:**
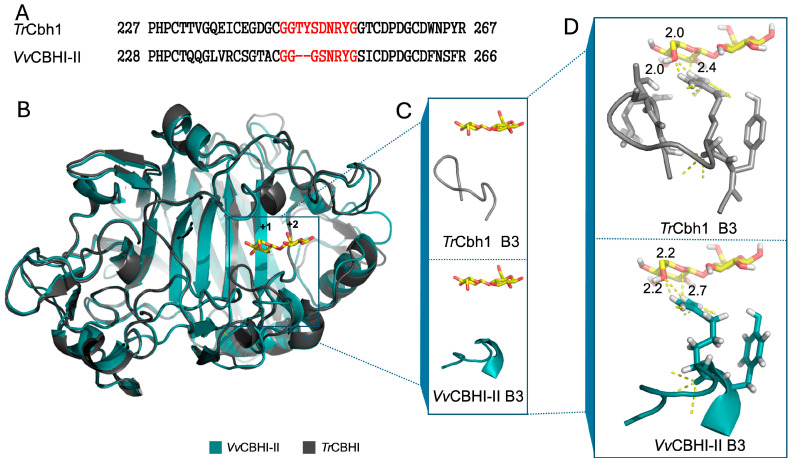
Structural determinants and hydrogen-bonding networks governing the low-temperature activity of *Vv*CBHI-II. (**A**) Local sequence alignment of the B3 loop region (shown in red) between *Tr*CBHI and *Vv*CBHI-II. (**B**) Overall 3D structural superposition of *Vv*CBHI-II and *Tr*CBHI. (**C**) Detailed structural comparison highlighting the replacement of the B3 loop with a *β*-sheet in *Vv*CBHI-II. (**D**) Magnified view of the product-binding subsites (+1 and +2) showing the altered hydrogen-bonding distances (indicated in Å) between the specific enzyme residues and the cellobiose product.

To elucidate the molecular mechanism underlying the outstanding low-temperature activity of *Vv*CBHI-II, we further investigated the structural distinctions at the product-binding subsites, building upon our previous structural homology models (Section 3.1). Sequence alignment (Figure 5A) and 3D structural overlays (Figure 5B,C) highlight the distinct truncation and structural substitution of the B3 loop in *Vv*CBHI-II. Intriguingly, a detailed analysis of the product binding sites (+1 and +2 subsites) reveals critical differences in the hydrogen-bonding networks between the enzyme and the cellobiose product (Figure 5D). In *Tr*CBHI, the extended B3 loop tightly embraces the product via short, strong hydrogen bonds with distances ranging from 2.0 to 2.4 Å. Conversely, in *Vv*CBHI-II, the structural variation of the B3 loop inherently expands the substrate-binding cleft, leading to elongated and significantly weakened hydrogen bonds (2.2 to 2.7 Å) with the cellobiose molecule.

This structural adaptation holds profound kinetic implications, particularly for cold-active catalysis [36]. In typical processive GH7 CBHs, the continuous hydrolysis of cellulose requires the sequential threading, cleavage, and expulsion of the product [37]. Previous biophysical studies have established that product dissociation—rather than the chemical cleavage itself—is the primary rate-limiting step, a bottleneck that is further exacerbated at low temperatures due to reduced thermodynamic kinetic energy [38,39]. The robust hydrogen-bonding network in *Tr*CBHI, while ensuring strict processivity, traps the cellobiose within the enclosed tunnel, leading to severe product inhibition under cold conditions. In striking contrast, the weakened hydrogen bonds and the more open cleft architecture governed by the modified B3 loop in *Vv*CBHI-II facilitate the rapid release of cellobiose from the catalytic channel [34]. This accelerated product dissociation effectively alleviates product inhibition and prevents the enzyme from stalling on the cellulose chain [31]. Consequently, *Vv*CBHI-II maintains a remarkably high catalytic turnover rate and robust synergistic degradation efficiency even in low-temperature environments, perfectly corroborating the EG-like hydrolytic performance observed in Section 3.3.

### 3.5. Low-Temperature Saccharification of Natural Lignocellulosic Biomass and Its Application in Juice Processing

**Figure 6:**
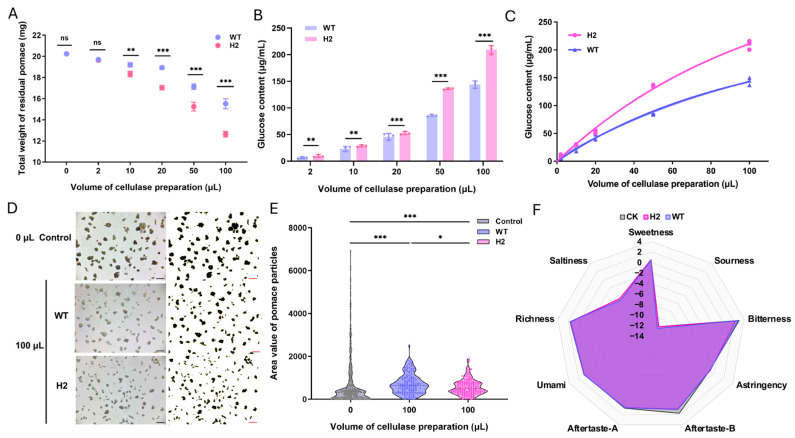
Low-temperature saccharification of pear pomace and its impact on juice sensory quality. (**A**) Residual dry weight of pear pomace after 12 h of enzymatic treatment at 10 °C with equivalent filter paper units of cellulase preparations from the wild-type (WT) and H2 transformant. (**B**) Corresponding glucose release during the saccharification process. (**C**) Non-linear fitting curves of the glucose release data using an exponential association model. (**D**) Macroscopic morphological changes of the residual pomace observed by means of stereomicroscopy. The images represent samples treated with 100 μL of the respective cellulase preparations (standardized at 10 FPU/mL). Scale bars = 1 mm. (**E**) Quantitative distribution of pomace particle areas analyzed via Image J. (**F**) Radar chart illustrating the E-tongue sensory evaluation of the juices treated with different enzyme preparations. (ns *p* > 0.05, * *p* < 0.05, ** *p* < 0.01, *** *p* < 0.001).

To evaluate the practical application potential of the engineered cold-active cellulase complex, natural lignocellulosic biomass derived from Dangshan pear pomace was subjected to low-temperature saccharification at 10 °C. The crude enzyme preparations from the H2 transformant and the WT strain were strictly standardized to an equivalent filter paper activity (10 U/mL at 50 °C) to ensure an impartial comparison. As illustrated in Figure 6A, following a 12 h incubation at 10 °C, the residual dry weight of the pomace treated with the H2 enzyme complex was significantly lower than that of the WT-treated group across all tested enzyme volumes (10–100 μL). Concurrently, the glucose released into the reaction system was quantified (Figure 6B). The H2 preparation exhibited a remarkably superior saccharification capacity, yielding substantially higher glucose concentrations than the WT preparation, with the disparity becoming increasingly pronounced at higher enzyme dosages.

To further elucidate the hydrolytic kinetics, the glucose release data were fitted to an exponential association model (Figure 6C). The fitting equations for the H2 and WT preparations were *Y_H_*_2_ = 343.8 * [1 − *e*^−0.009502*x^] (*R*^2^ = 0.9943) and *Y_WT_* = 218.7 * [1 − *e*^−0.01058*x^] (*R*^2^ = 0.9906), respectively. The predicted maximum theoretical glucose release (*Y_max_*) for the H2 strain was 343.8 μg/mL, which is approximately 1.57-fold higher than that of the WT strain (218.7 μg/mL). This mathematical projection firmly confirms that the incorporation of *Vv*CBHI-II endows the cellulase complex with a profoundly enhanced hydrolytic limit on natural biomass under cold conditions.

Furthermore, to comprehensively evaluate the absolute completeness of this enzymatic hydrolysis, the polymer composition of the raw material must be considered. Based on previous compositional analyses, the dry matter of pear pomace typically comprises approximately 15–25% cellulose, 10–20% hemicellulose, and 10–15% pectin [40]. Assuming an average cellulose content of 20%, the absolute theoretical maximum glucose yield is approximately 222 mg per gram of dry biomass (accounting for the hydration factor of 1.11). By converting our kinetic *Y_max_* (343.8 μg/mL in the 2 mL reaction system containing 0.02 g of pomace) to the equivalent yield per gram of dry pomace, which equates to 34.4 mg/g, the estimated maximum hydrolysis conversion efficiency achieved by the H2 complex is roughly 15.5%. While a portion of the recalcitrant polysaccharides inevitably remains trapped within the highly lignified stone cell structures without thermochemical pretreatment, this compositional conversion rate represents a substantial improvement over the wild-type enzyme, satisfying the industrial prerequisite for efficient low-temperature depolymerization.

The macroscopic degradation of the pomace was visually corroborated using stereomicroscopy (Figure 6D and Figures S2–S5). As depicted in Figure 6D, after 12 h of enzymatic hydrolysis (using 100 μL of the preparations), the pomace particles treated with the H2 enzyme were visibly more fragmented and structurally disrupted compared to the control and WT-treated samples. Given that macroscopic visual differences can be subtle, a rigorous quantitative analysis of the particle area distribution using Image J was performed (Figure 6E) to statistically validate these observations. The average particle area of the H2-treated pomace was significantly reduced, exhibiting a highly concentrated distribution of smaller particles. This pronounced morphological disruption perfectly aligns with our earlier structural conclusions: *Vv*CBHI-II, with its unique open active-site cleft, exhibits robust EG-like behavior that effectively depolymerizes complex lignocellulosic matrices and alleviates product inhibition even at low temperatures.

Finally, to simulate actual fruit juice processing, 200 g of homogenized Dangshan pear was treated with 4 FPU of the respective enzyme preparations at 10 °C for 24 h. Consistent with the in vitro saccharification assay, the dry matter (DM) of the residual filtered pomace was lowest in the H2-treated group, followed by the WT and the untreated control (CK) (Figure S6). Furthermore, the flavor profile of the resulting juice was quantitatively evaluated using an E-tongue system (Figure 6F). Notably, the sweetness response value of the juice treated with the H2 enzyme complex was significantly enhanced to 0.55 ± 0.05, outperforming both the WT group (0.46 ± 0.02) and the CK group (0.34 ± 0.05) (Table S3). These results unequivocally demonstrate that the cold-active glycoside hydrolase system containing *Vv*CBHI-II not only maximizes juice extraction yield by minimizing waste residue but also significantly improves the sensory quality of the juice by efficiently liberating soluble sugars and flavor precursors under temperature-sensitive processing conditions.

Furthermore, in addition to the remarkable enhancement in sweetness, the incorporation of the *Vv*CBHI-II engineered complex profoundly optimized the broader sensory profile of the juice by mitigating undesirable flavor attributes. As detailed in Table S3, the juice treated with the H2 preparation exhibited a significantly reduced bitterness score (2.32 ± 0.10) compared to both the WT-treated (2.88 ± 0.11) and the untreated CK (3.03 ± 0.20) groups. Crucially, the persistent off-flavors—specifically the aftertastes associated with bitterness (Aftertaste-B) and astringency (Aftertaste-A)—were substantially suppressed in the H2-treated samples, reaching the lowest recorded values of 0.82 ± 0.03 and 0.52 ± 0.01, respectively. Concurrently, the application of this cold-active cellulase system led to a striking reduction in residual pomace (Figure 6A and Figure S6), demonstrating its robust capability to depolymerize the insoluble plant cell wall matrix into soluble components.

From a broader industrial perspective, the deployment of such highly efficient cold-active cellulases represents a transformative approach for the fruit juice industry and food waste biorefineries. Enzymatic treatments using tailored carbohydrases are increasingly recognized as an eco-friendly and sustainable strategy to valorize fruit byproducts; they facilitate the maximal recovery of fermentable sugars and bioactive compounds while simultaneously improving the sensory qualities of the final products [41]. Therefore, the development and application of customized, low-temperature glycoside hydrolases like *Vv*CBHI-II will not only maximize juice extraction yields and minimize solid waste disposal, but also pave the way for more energy-efficient, zero-waste, and premium-quality fruit processing paradigms in the circular bioeconomy [42].

## 4. Conclusions

This study successfully developed a highly efficient, cold-active cellulase system by integrating the novel *V. volvacea* cellobiohydrolase *Vv*CBHI-II into *T. reesei*. The exceptional low-temperature catalytic performance of *Vv*CBHI-II is fundamentally governed by a structural truncation in its B3 loop, which expands the active-site cleft. This unique architecture weakens product-enzyme hydrogen bonds, accelerating product dissociation to circumvent cold-induced product inhibition while conferring robust EG-like hydrolytic flexibility. Consequently, the engineered cellulase complex demonstrated a profoundly enhanced saccharification limit on natural lignocellulosic biomass under cold conditions. Applied to fruit juice processing, it efficiently depolymerized waste pomace, maximized soluble sugar release, and significantly optimized the overall sensory profile. Ultimately, the discovery and application of *Vv*CBHI-II present a transformative, eco-friendly biocatalytic strategy for temperature-sensitive food processing and zero-waste biomass valorization.

## Figures and Tables

**Table 1:** Substrate specificities of cellobiohydrolases towards cellulosic substrates.

Enzymes	Specific Activity (U/mg)				
	PASC	Avicel	CMC-Na	Filter Paper	*p*NPC
*Vv*CBHI-II	3.321 ± 0.056 ^a^	0.136 ± 0.016 ^b^	0.234 ± 0.024 ^a^	0.356 ± 0.038 ^a^	0.083 ± 0.011 ^a^
*Vv*CBHI-I	1.231 ± 0.012 ^b^	0.265 ± 0.023 ^a^	0.081 ± 0.002 ^b^	0.183 ± 0.005 ^b^	0.035 ± 0.005 ^b^
*Tr*CBHI	0.218 ± 0.002 ^c^	0.231 ± 0.023 ^ab^	0.042 ± 0.002 ^c^	0.125 ± 0.003 ^c^	0.025 ± 0.002 ^c^

Values represent the mean ± standard deviation of triplicates. Different superscript letters (a, b, c) within the same column indicate statistically significant differences (*p* < 0.05) according to Duncan’s multiple range test.

## Data Availability

The original contributions presented in this study are included in the article/Supplementary Material. Further inquiries can be directed to the corresponding authors.

## Supplementary Materials

The following supporting information can be downloaded at: https://www.mdpi.com/article/10.3390/jof12040276/s1, Table S1. Primers used in this study. Table S2. Codon-optimized DNA sequence of VVO_07197. Table S3. E-tongue results for cellulase preparation treated pear juice. Data S1. The re-annotated GH7 family glycoside hydrolases from Volvariella volvacea. Figure S1. Schematic diagram of the homologous recombination (HR) plasmid construction (plasmid backbone omitted). Figure S2. Low-temperature saccharification of pear pomace with 2 μL of the respective cellulase preparations. Figure S3. Low-temperature saccharification of pear pomace with 10 μL of the respective cellulase preparations. Figure S4. Low-temperature saccharification of pear pomace with 20 μL of the respective cellulase preparations. Figure S5. Low-temperature saccharification of pear pomace with 50 μL of the respective cellulase preparations. Figure S6. Dry matter (DM) of the residual filtered pear pomace after low-temperature enzymatic treatment.

## Abbreviations

The following abbreviations are used in this manuscript:

DNS	3,5-dinitrosalicylic acid
HR	Homologous recombination
IU	International unit
PASC	Phosphoric acid-swollen cellulose
*p*NP	*p*-nitrophenol
