# Supplementary material for: Engineering a Cold-Active Cellulase Complex with a Novel Mushroom Cellobiohydrolase for Efficient Biomass Saccharification and Juice Flavor Optimization
Source: J Fungi (Basel). 2026 Apr 10;12(4):276. doi: 10.3390/jof12040276 (PMC13118234; doi:10.3390/jof12040276)
Supplement: Supplementary file 1 [file jof-12-00276-s001.zip › jof-4227087-supplementary.pdf]

Table S1. Primers used in this study.

Primers	Sequences 5’→3’	Usage
VVO_07197F	caatagtcaaccgcggactgcgcatcatgtttcccgccgccacc	Amplification and verification for *vvcbhI-II* gene
VVO_07197R	gatcccggtcggcatctactttaatggtgatggtgatgatgggcgctgtaggtagaacc	
HygF	ctatgaaaattccgtcaccagccctgggatccgcgcaattaaccctcac	Amplification for *HygB*
HygR	cagggctggtgacggaattttcatag	
TtrC1F	agtagatgccgaccgggatcgatc	Amplification for the terminator T_{trpC}
TtrC1R	cagggctggtgacggaattttcatag	
Pcbh1F	cggccagtgccaagcttgcatgcctgcaggctccgaagctgctgcgaacccgg	Amplification and verification for promoter P_{cbh1}
Pcbh1R	gatgcgcagtccgcggttgac	
Pcbh107197F	cggccagtgccaagcttgcatgcctgcaggctccgaagctgctgcgaacccgg	Amplification for the 5’ flanking sequence
Pcbh107197R	gttaggagaaggctgccaaccctggac	
TtrpC1-HygF	ctatgaaaattccgtcaccagccctgggatccgcgcaattaaccctcac	Amplification for the 3’ flanking sequence
TtrpC1-HygR	ggggctgatctgaccagttgcc	
cbh1F	gtatcggaagttggccgtcatctcg	Verifying HR on *trcbh1* locus
cbh1R	gtctcgtttgtcgggtaggtggag	
UpF	cctaaaccgaagtgcgtggtaaatctacacg	Verifying HR on 5’ flanking of *trcbh1* locus
VVO_07197R	gatcccggtcggcatctactttaatggtgatggtgatgatgggcgctgtaggtagaacc	
TtrCF	agtagatgccgaccgggatcgatc	Verifying HR on 3’ flanking of *trcbh1* locus
DnR	gccagctatggtctcactgtgttc	

Table S2. Codon-optimized DNA sequence of VVO_07197.

Gene	Sequence 5’→3’
VVO_07197	atgtttcccgccgccaccctctttgccttttctctctttgccgccgtctacggtcagcaggtcggtacccagctcgccgagacccaccctcgactcacctggcagaagtgc<br>acccgaagcggcggttgccagacccagtctaacggcgccattgtcctcgacgccaattggcgctgggtccacaacgtcggcggttacaccaattgctacaccggtaa<br>cacctggaatacctctctctgccccgacggcgccacctgcgccaagaattgcgccctcgacggcgccaattaccagtctacctacggtattaccaccagcggtaacgc<br>cctcaccctcaagtttgtcacccagagcgagcagaagaacatcggttctcgagtctacctcctcgagtccgacaccaagtaccagctctttaaccccctcaaccaggag<br>tttacctttgacgtcgacgtctctcagctcccttgcggtctcaacggcgccgtctacttttctgccatggacgccgacggcggcatgtctaagtttcctaacaacgccgccg<br>gcgccaagtacggtaccggttactgcgactctcagtgccctcgagacatcaagttcattaacggcgaggccaacgtccagggttggcagccttctcctaacgacacca<br>acgccggtaccggtaattacggcgcctgctgcaacgagatggacgtctgggaggccaactctatttctaccgcctacacccctcacccttgcacccagcagggtctcg<br>tccgatgcagcggtaccgcctgcggcggcggttctaatcgatacggttctatttgcgaccccgacggttgcgactttaactctttccgcatgggcgacaagagcttttacg<br>gtcccggtctcaccgtcaatacccagcagaagtttaccgtcgtcacccagttcctcaccaacaacaactcttctagcggtaccctccgagagattcgacgactctacgtc<br>cagaacggtcgagtcattcagaacagcaaggtcaacattcccggtatgccttctaccatggactctgtcaccaccgagttttgcaacgcccagaagaccgcctttaacg<br>acaccttcagctttcagcagaagggcggtatggccaatatgagcgaggccctccgacgaggtatggtcctcgtgctctctatttgggacgaccacgccgccaatatgct<br>ctggctcgactctaattaccctaccgaccgacctgcctctcagcctggtgtcgcccgaggtacctgccctacctctagcggtaagcctagcgacgtcgagaattctacc<br>gccaactctcaggtcatctacagcaacatcaagttcggcgacattggttctacctacagcgcccatcatcaccatcaccattaa

Table S3. E-tongue results for cellulase preparation treated pear juice

Samples	Sweetness	Sourness	Bitterness	Astringency	Aftertaste-B	Aftertaste-A	Umami	Richness	Saltiness
CK	0.34 ± 0.05^{c}	-11.69 ± 0.64^{a}	3.03 ± 0.20^{a}	-1.02 ± 0.04^{a}	1.67 ± 0.09^{a}	0.66 ± 0.02^{a}	0.68 ± 0.02^{b}	1.52 ± 0.08^{a}	-4.68 ± 0.04^{a}
H2	0.55 ± 0.05^{a}	-11.74 ± 0.60^{a}	2.32 ± 0.10^{b}	-0.96 ± 0.02^{a}	0.82 ± 0.03^{c}	0.52 ± 0.01^{c}	0.78 ± 0.01^{a}	1.64 ± 0.03^{a}	-4.73 ± 0.01^{a}
WT	0.46 ± 0.02^{b}	-12.17 ± 0.73^{a}	2.88 ± 0.11^{a}	-0.99 ± 0.08^{a}	0.93 ± 0.04^{b}	0.58 ± 0.02^{b}	0.75 ± 0.05^{ab}	1.70 ± 0.09^{a}	-5.08 ± 0.04^{b}

Values represent the mean ± standard deviation of triplicates. Different superscript letters (^{a}, ^{b}, ^{c}) within the same column indicate statistically significant differences ($p < 0.05$) according to Duncan’s multiple range test.

Data S1. The re-annotated GH7 family glycoside hydrolases from *Volvariella volvacea*

>VVO_03251H
MFPKSSLLVLSFLATAYAQQVGTVTAETHPSLSWSRCTSSGCQSVAGSVTLDANWRWLHTTEGYTNCYTGNSWDTSLCPDGAT
CAQNCALDGANYQSTYGITTSGDALTLKFITQGEQKNVGSRVYLMASESQYEMFELLNKEFTFDVDVSNLPCGLNGALYFSSM
DADGGMAKNSGNKAGAKYGTGYCDSQCPRDIKFINGEANVAGWNGSPNDTNAGTGNWGACCNEMDIWEANSISTAYTPHPC
TVQGLSRCEGSSCGTNDRYGTVCDPDGCDFNSYRMGDKTYYGPGGTGVDTRSKFTVVTQFFTNNNSSSGTLSEIRRLYVQNGQ
VVQNSKVNIPGMDAYDSITGEFCDSQKSVFGDTTSFQNKGGMQAMGQALGAGMVLVLSVWDDHAANMLWLDSSYPVDADP
NQPGIARGTCPTDSGKPIDVEESAANASVTFSNIRFGDIGTTYTGGSTTPGNPGNPGTTTTTAPGPVQTKWGQCGGQGWTGPTQ
CESGSTCSVLNEWYSQCL

>VVO_06238H
MFRKAALIAFSFFSIAYGQQVGTVQAENHPRLTWSKCTASGCVAQSSGSVVLDANWRWVHDKNGYTNCYTGNAWNTTICPNG
STCASNCALDGADYQGTYGITTSGNSLTLKFVTQSSQKNVGSRVYLLASDTRYEMFNLLNKEFTFDVDVSQLPCGLNGALYFSE
MDADGGTSRFPTNTAGARYGTGYCDSQCARDIKFINGEANVANWTASPTDPNAGTGQYGACCGEMDIWEANSISTAYTPHPCT
STGQVRCSGTACGGPNNRYGSVCDPDGCDFNSYRQGVYDFYGPGSSNTIDTTKKVTVVTQFYTSNNSTTGTLSEIRRIYVQDGR
VIQNSKTTFSGISPYDSITSQYCADQKTFFNDHESFASKGGMAGMGRALARGMVLVMSIWDDHYAHMLWLDSTYPTDADPSIP
GKKRGTCPTTSGDPKDVEAQYPNAQVTFSNIRFGDIGTTYTGGGSTSNPGTPTTTTSAPAATQTRWGQCGGQGWTGPTACESG
TRCVVVNQWYHQCQ

>VVO_03244H
MFPKSTSFVIIGVLSTAYCQQAGTVVPETHPSLSWSRCNSTGCQSVVASIVLDANWRWIHNVAGYTNCYAGNTWNTTLCPEGTI
CASNCALDGADYQATYGISTSGNSITLKFNGNMRQNLGSRVYLLNTETRYEIFQLLNKELTFDVDASNLPCGFNGALYFTSMDA
DGGMAKYPGNKAGAKYGTGYCDSKCPRNLKFINGEANVQGWTPSPQDPNSGTGYYGSCCNEMDIWEANSISAVYTLHPCTV
QGPSRCSGTACGINDRYGTTCDPDGCDFNSFRMGDPYFYGPGGTSINSQHKITVVTQFLTNNNSSTGTISEIRRLYIQNGHVIQNS
KVNIPEMNNTLDSITGAFCDAQKTAFGDPRSFQDKGGMEAMGRALGGGMVLTMSIWKDPERDMLWLDSSYPLDADPSKPGV
RRGTCGPNTGPTIPEDPDDQRVTFSNIRFGDIGTTYTAA

>VVO_06080H
MFRKAALLSLAYAALSYAQQVGTVVAENHPRLSWQQCTTSGGCRTASTGSITLDANWRWVHTTSGYTNCYTGSSWNSTICPN
GATCAQNCALDGAEYASTYGITTNGDSLTLKFVTTSGGSKNVGGRVYLMNSENRYEMFQVLNKEFTFDVDVSQLPCGLNGAL
YFVEMEADGGSGRFPSNRAGAKYGTGYCDSQCPRDIKFINGEANVAGWSASPTDPNAGTGNWGACCAEMDIWEANSISNAYT
PHPCTQPGLTRCTDCTSGGRYGTVCDPDGCDFNPYRQGDRDFYGPGSSNTIDTTKKVTVVTQFFTHDNTTTGRLTEIRRLYVQD
GRVIQNTRTDFSGIDPYDSITSDYCADQKAFFNDHESFGSKGGMQAMGESLRRGHVLVMSIWNDHGAHMLWLDSTYPTDADP
NVPGKARGTCPTNSGVPAETEANYPNAQVTFSNIRFGDIGSTYGAGTGPSNPGTPTSTSTAPSATQTRWGQCGGQGWSGPTVCE
SGTTCNVINQWYHQCI

>VVO_03243
MTNGQQIGTISPETHPPLSWSRCNSTGCQPVAASIVLDANWRWIHNTGGYTNCYTGNTWNTTLCPEGTICASNCALEGADYRS
TYGITTSGNSITLVFDEEYRRSLGSRVYLLNTETRYEVFQLLNKEFTFDVDVSDLPCALNGALYFTSMDADGGMTKYPANKAGA
KYGTGYCDSKCPRNVEFIYGEANVQGWTPSPNDPNSGTGYYGSCCNEMDIWEANSISAVYTLHPCTVQGPSRCSGTACGTNNR
YGTVCDPDGCDFNSYRMGNSSFYGFGGTGVNTRSNSTGTLSEIRRLYIQNGRVIQNSKVNIPGMSNTLDSITSAFCDAQKTAFGD
PRSFQDKGGMEAMGRALGSGMVLAMGISKDYESNMLWLDGSFLFDADPSKPGVRRSICRPTDPYDPNLPVLQYLKGTVTFSNI
KFGDIGSTYATV

>VVO_03230
MFPKSSLLFLSFLATAYAQQVGTQTAEVHPSLNWARCTSSGCTNVAGSVTLDANWRWLHTTSGYTNCYTGNTWNTTLCPDGAT
CAQNCALDGASYQSTYGITTSGNALTLKFVTQSAQKNIGSRVYLMASDTQYEMFQLLNKEFTFDVDVSNLPCGLNGALYFSSM
DADGGMAKYPSNKAGAKYGTGYCDSQCPRDIKFINGEANVAGWVGSPNDTNAGTGNWGACCNEMDIWEANSISAAYTPHPC
TVQGLSRCSGTACGTNDRYGTVCDPDGCDFNSYRMGDKTYYGPGGTGVDTRSKFTVVTQFLTNNNSSSGTLSEIRRLYVQNGQ
VVQNSKVNIPGMSAYDSITGAFCDAQKTAFGDTRSFQNKGGMSAMGQALGTGMVLVLSIWDDHAANMLWLDSNYPVDADPS
KPGIGRGTCPTTSGNPSDVEVSAANSSVTFSNIKFGDIGTTYTGGSVTTPGTTSGTTTSTAPGAVQTKWGQCCLFFSVSGGQGWS
GPTQCESGSTCTVVNQWYSQCI

>VVO_04567
MFPKAALISFSLFAIAYGQQVGTAKTEVHPKLSWQKCTTSGGCVTQSTGEVTLDSNWRWVHDKNGYTNCYTGNAWNSTICPD
GVTCANNCAVDGADYQATYGITTSGNSLTLKFVTQSSQKNVGSRVYLMNSESRYEMFNLLNKEFTFDVDVSQLPCGLNGALYF
VEMDADGGMSRFSGNKAGAKYGTGYCDSQCPRDIKFINGEANVAGWSASPTDPNAGTGNYGTCCGEMDIWEANSISTAYTPH
PCTSTGQVRCTGNACGGSSNRYGSVCDPDGCDFNSYRQGVYNFYGPGSSNTIDTNKKVTVVTQFFTNNNSSTGTLTEIRRLYVQ
DGRVIQNSKTSFSGISPYDSITSQYCADQKSFFGDHESFASKGGLARMGAAIARGVVLVMSIWDDHYAHMLWLDSTYPTDADPS
VPGKKRGTCPTTSGDPKDVEANSANAQVVFSNIRFGDIGSTYSGGSTSNPPTSTTTSTAPGPTQTQWGQCGGQGWTGPTVCQT
GTRCVVVNQWYHQCQ

>VVO_03260
MFPKSSLLVLSFLATAYAQQVGTQTAEVHPSLNWARCTSSGCTNVAGSVTLDANWRWLHTTSGYTNCYTGNSWNTTLCPDGAT
CAQNCALDGANYQSTYGITTSGNALTLKFVTQGEQKNIGSRVYLMASESRYEMFSLLNKEFTFDVDVSNLPCGLNGALYFSSM
DADGGMAKNPGNKAGAKYGTGYCDSQCPRDIKFINGEANVAGWNGSPNDTNAGTGNWGACCNEMDIWEANSISAAYTPHP
CTVQGLSRCSGTACGTNDRYGTVCDPDGCDFNSYRMGDKTYYGPGGTGVDTRSKFTVVTQFLTNNNSSSGTLSEIRRLYVQNG
RVVQNSKVNIPGMSNTLDSITTGFCDSQKTAFGDTRSFQNKGGMSAMGQALGAGMVLVLSVWDDHAANMLWLDSNYPVDA
DPSKPGIARGTCSTTSGKPTDVEQSAANSSVTFSNIKFGDIGTTYTGGSVTTTPGNPGTTTSTAPGAVQTKWGQCGGQGWTGPT
RCESGSTCTVVNQCF

>07197
MFPAATLFAFSLFAAVYGQQVGTQLAETHPRLTWQKCTRSGGCQTQSNGAIVLDANWRWVHNVGGYTNCYTGNTWNTSLCP
DGATCAKNCALDGANYQSTYGITTSGNALTLKFVTQSEQKNIGSRVYLLESDTKYQLFNPLNQEFTFDVDVSQLPCGLNGAVYF
SAMDADGGMSKFPNNAAGAKYGTGYCDSQCPRDIKFINGEANVQGWQPSPNDTNAGTGNYGACCNEMDVWEANSISTAYTP
HPCTQQGLVRCSGTACGGGSNRYGSICDPDGCDFNSFRMGDKSFYGPGLTVNTQQKFTVVTQFLTNNNSSSGTLREIRRLYVQN
GRVIQNSKVNIPGMPSTMDSVTTEFCNAQKTAFNDTFSFQQKGGMANMSEALRRGMVLVLSIWDDHAANMLWLDSNYPTDR
PASQPGVARGTCPTSSGKPSDVENSTANSQVIYSNIKFGDIGSTYSA

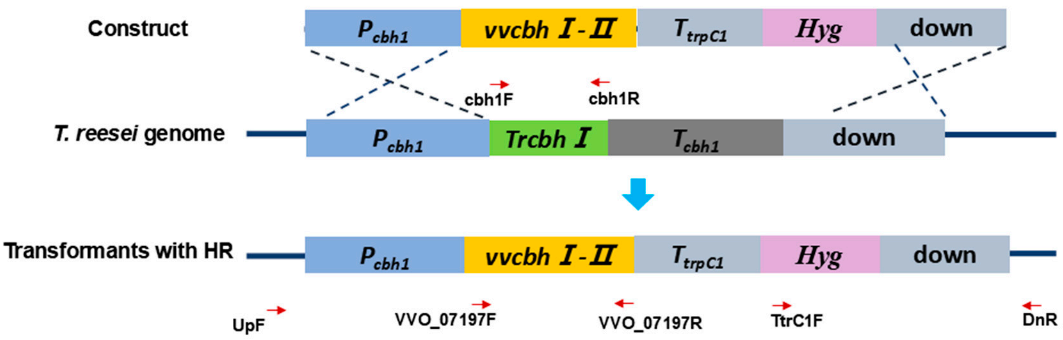

Figure S1. Schematic diagram of the homologous recombination (HR) plasmid construction (plasmid backbone omitted). Following homologous recombination between the construct and the tr*cbh1* locus in the T. reesei genome, transformants are identified as HR transformants if PCR results are positive for the primers indicated by arrows (VVO_0719F/R, UpF/VVO_07197R, and TtrC1F/DnR) and negative for cbh1F/R. Conversely, transformants are identified as NHEJ (Non-Homologous End Joining) transformants if PCR results are positive for VVO_0719F/R and cbh1F/R, but negative for UpF/VVO_07197R and TtrC1F/DnR.

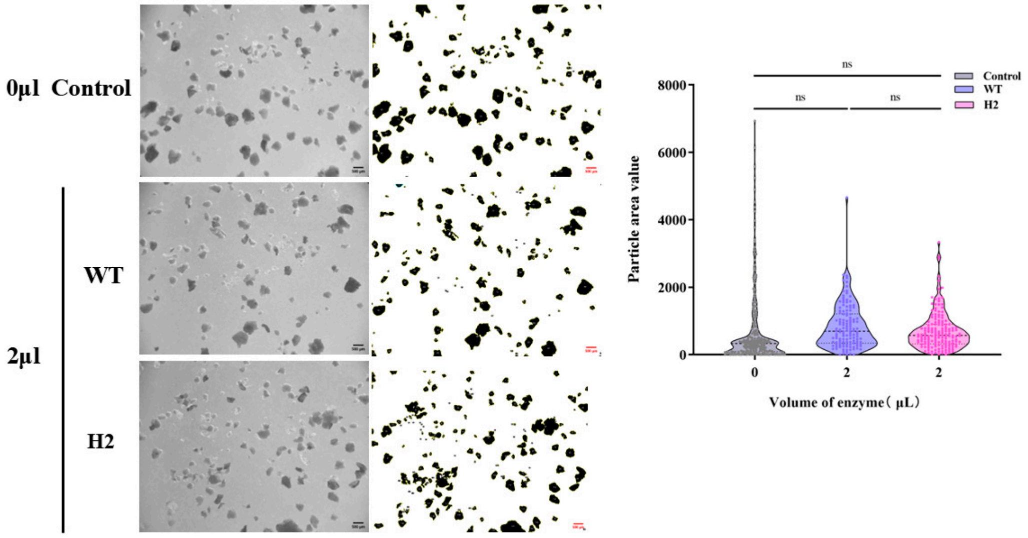

Figure S2. Low-temperature saccharification of pear pomace with 2 μL of the respective cellulase preparations. Macroscopic morphological changes of the residual pomace (2 μL cellulase complex standardized at 10 FPU/mL) observed by stereomicroscopy. Scale bars = 500 μm. (ns $p > 0.05$)

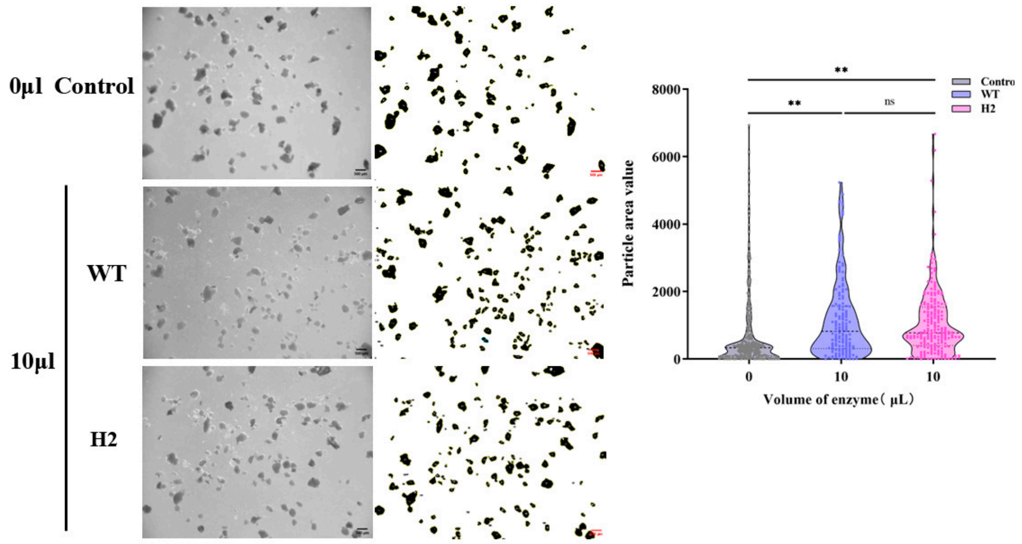

Figure S3. Low-temperature saccharification of pear pomace with 10 µL of the respective cellulase preparations. Macroscopic morphological changes of the residual pomace (10 µL cellulase complex standardized at 10 FPU/mL) observed by stereomicroscopy. Scale bars = 500 µm. (ns $p > 0.05$, ** $p < 0.01$)

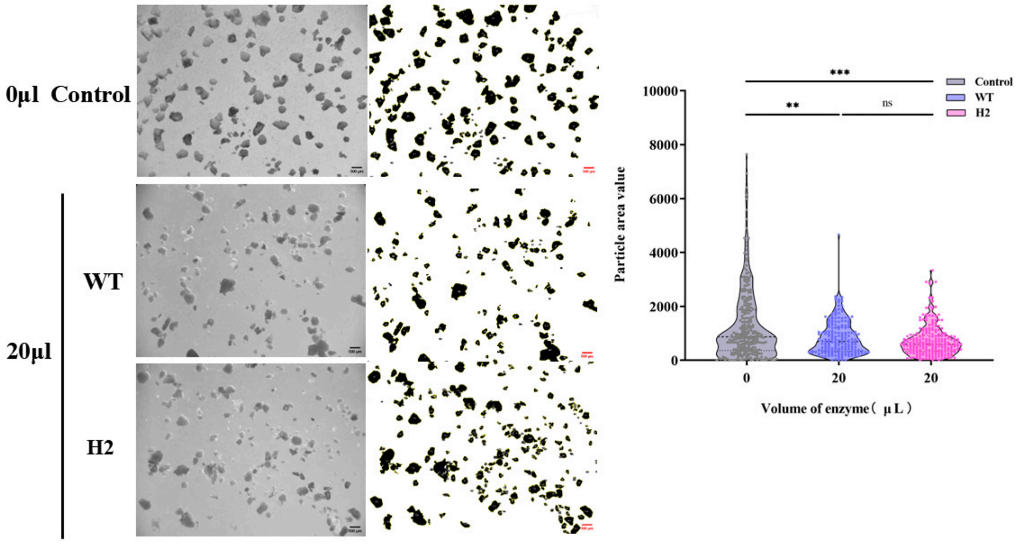

Figure S4. Low-temperature saccharification of pear pomace with 20 μL of the respective cellulase preparations. Macroscopic morphological changes of the residual pomace (20 μL cellulase complex standardized at 10 FPU/mL) observed by stereomicroscopy. Scale bars = 500 μm. (ns $p > 0.05$, ** $p < 0.01$, *** $p < 0.001$).

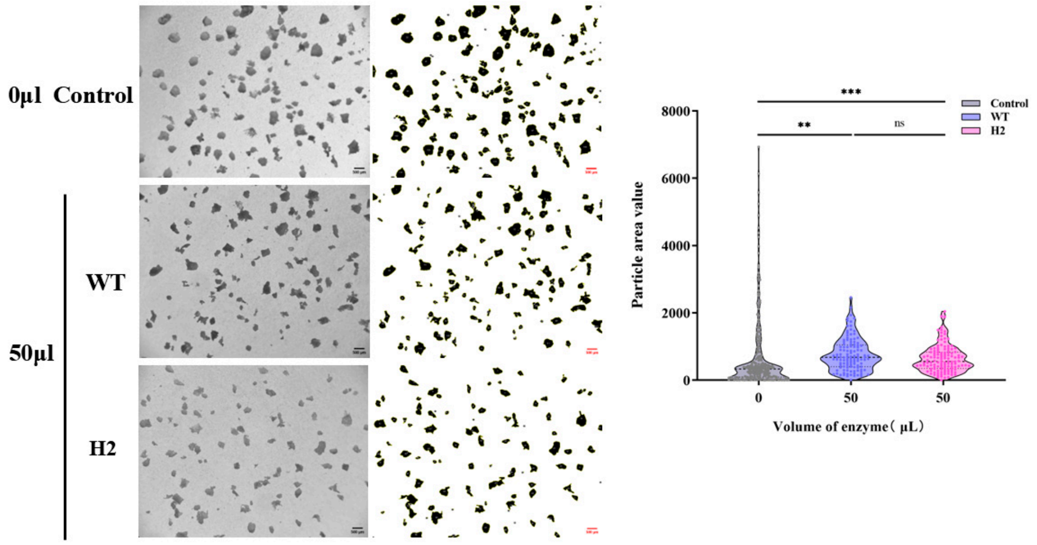

Figure S5. Low-temperature saccharification of pear pomace with 50 μL of the respective cellulase preparations. Macroscopic morphological changes of the residual pomace (50 μL cellulase complex standardized at 10 FPU/mL) observed by stereomicroscopy. Scale bars = 500 μm. (ns $p > 0.05$, ** $p < 0.01$, *** $p < 0.001$).

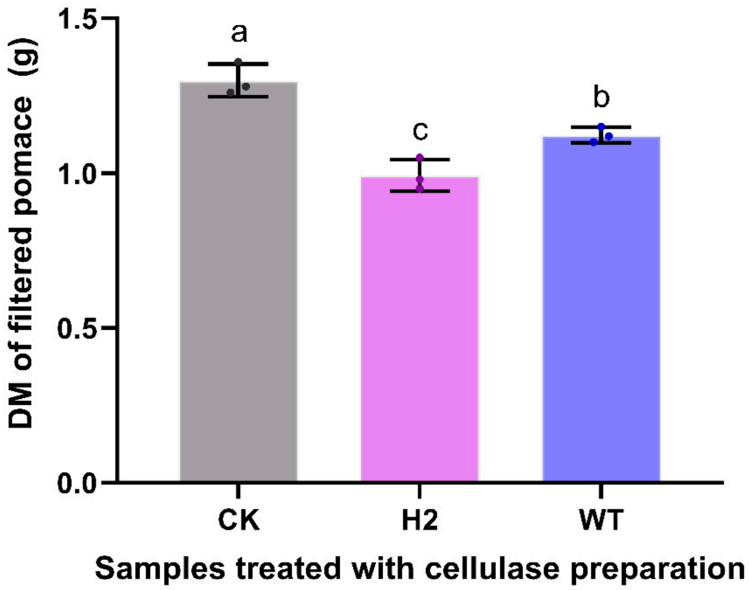

Figure S6. Dry matter (DM) of the residual filtered pear pomace after low-temperature enzymatic treatment. Homogenized Dangshan pear (200 g) was treated with 4 FPU of crude cellulase preparations derived from the engineered H2 transformant and the wild-type (WT) strain at 10 °C for 24 h. The untreated sample served as the control (CK). After incubation, the mixtures were filtered through double-layer gauze, and the residual pomace was dried to a constant weight. Data are presented as the mean ± standard deviation of replicates. Different superscript letters (a, b, c) within the same column indicate statistically significant differences ($p < 0.05$) according to Duncan's multiple range test.
